# The Neuromodulatory Basis of Aggression: Lessons From the Humble Fruit Fly

**DOI:** 10.3389/fnbeh.2022.836666

**Published:** 2022-04-18

**Authors:** Caroline B. Palavicino-Maggio, Saheli Sengupta

**Affiliations:** ^1^Basic Neuroscience Division, Department of Psychiatry, Harvard Medical School, McLean Hospital, Boston, MA, United States; ^2^Department of Neurobiology, Harvard Medical School, Boston, MA, United States

**Keywords:** neuromodulator, aggression, serotonin, acetylcholine, dopamine, octopamine, peptides, *Drosophila melanogaster*

## Abstract

Aggression is an intrinsic trait that organisms of almost all species, humans included, use to get access to food, shelter, and mating partners. To maximize fitness in the wild, an organism must vary the intensity of aggression toward the same or different stimuli. How much of this variation is genetic and how much is externally induced, is largely unknown but is likely to be a combination of both. Irrespective of the source, one of the principal physiological mechanisms altering the aggression intensity involves neuromodulation. Any change or variation in aggression intensity is most likely governed by a complex interaction of several neuromodulators acting via a meshwork of neural circuits. Resolving aggression-specific neural circuits in a mammalian model has proven challenging due to the highly complex nature of the mammalian brain. In that regard, the fruit fly model *Drosophila melanogaster* has provided insights into the circuit-driven mechanisms of aggression regulation and its underlying neuromodulatory basis. Despite morphological dissimilarities, the fly brain shares striking similarities with the mammalian brain in genes, neuromodulatory systems, and circuit-organization, making the findings from the fly model extremely valuable for understanding the fundamental circuit logic of human aggression. This review discusses our current understanding of how neuromodulators regulate aggression based on findings from the fruit fly model. We specifically focus on the roles of Serotonin (5-HT), Dopamine (DA), Octopamine (OA), Acetylcholine (ACTH), Sex Peptides (SP), Tachykinin (TK), Neuropeptide F (NPF), and Drosulfakinin (Dsk) in fruit fly male and female aggression.

## Introduction

Animals display aggression to acquire food, territories, and mating partners (Sturtevant, 1915; Hoffmann, 1987; Lin et al., 2011; Kravitz and Fernandez, 2015; Asahina, 2017; Anderson, 2016; Palavicino-Maggio et al., 2019a). However, the intensity of aggression must be modulated in accordance with changes in the external environment (such as, quality of the resource, size of the competitor, etc.) as well as internal environment (such as internal state, metabolic demands, etc.) (Lim et al., 2014; Li-Byarlay et al., 2014; Anderson, 2016; Asahina, 2017). An innate behavior such as aggression is encoded by genetically hardwired neural circuits. A critical question is, how does a genetically hardwired circuit allow flexible outputs of the same behavior? In other words, how are different intensities of the same behavior, in this case aggression, computed at the circuit-level? One of the principal mechanisms allowing such behavioral flexibility is neuromodulation (Bargmann, 2012; Bargmann and Marder, 2013; Kim et al., 2017). Neuromodulators are signaling molecules released from neuronal processes, which may alter circuit outputs by modulating the biochemical and electrophysiological properties, metabolic demands, and transcriptional profile of target neurons. Neuromodulators communicate with target neurons via synaptic transmission and/or volume transmission (Civelli, 2012; Marder, 2012; Nadim and Bucher, 2014; Asahina, 2017). Synaptic transmission is a form of point-to-point transmission of neuromodulators between anatomically proximal neurons. Volume transmission, on the other hand, is a form of extra-synaptic mode of transmission in which neuromodulators may be released in a diffuse manner from neuronal endings with the potential to communicate with anatomically distant neurons (Uzelac, 1998; Bucher and Marder, 2013; Nadim and Bucher, 2014; Taber and Hurley, 2014). Compared to volume transmission, synaptic transmission has received and continues to receive more research attention (Taber and Hurley, 2014). Unlike the fast-acting neurotransmitters or gap-junctions, neuromodulation occurs with relatively slower kinetics, over longer time scales, and is well suited to encode persistent behaviors such as aggression (Yurkovic et al., 2006; Kim et al., 2017, 2018; McCormick et al., 2020).

Classical studies in the invertebrate models such as those on the circuit dynamics of chemosensory behaviors in *Caenorhabditis elegans* or stomatogastric nervous system-mediated rhythmic motor pattern generation in crabs and lobsters, have shed light on the myriad ways by which neuromodulators might modify the composition and function of activated neuronal circuits and in effect, modify the outputs of a behavior (Bargmann, 2012; Marder, 2012; Bargmann and Marder, 2013). The fruit fly model of *Drosophila melanogaster* has also been a forerunner in elucidating the neuromodulatory basis of many social behaviors. Findings from the fruit fly model have provided deep, mechanistic understanding of how neuromodulators and their receptors interact within a circuit to modulate aggression (Kravitz and Fernandez, 2015; Asahina, 2017), sleep (Artiushin and Sehgal, 2017; Shafer and Keene, 2021), memory (Margulies et al., 2005), courtship (Greenspan and Ferveur, 2000), locomotion (Clark et al., 2018), etc. In this review, we highlight the current research findings on aggression from the fruit fly model, note findings from the mammalian models for comparison, and speculate on future direction of research. We focus on the aggression-regulatory roles of Serotonin (5-HT), Dopamine (DA), Octopamine (OA), Acetylcholine (ACTH), Sex Peptide (SP), Tachykinin (TK), Neuropeptide F (NPF), and Drosulfakinin (Dsk). It is worth noting that majority of these neuromodulators have primarily been researched in the context of male aggression. Female aggression, on the other hand, is far less known. This review summarizes the current state of knowledge for both male and female aggression in the fruit fly model for each of these neuromodulators.

Systematic analysis of female aggression has had a slow start compared to males. While male aggression has been studied in many species from the 1900’s (Sturtevant, 1915), little research was done to understand the neural mechanisms governing female aggression. It is difficult to pinpoint the reasons behind the discrepancy of interest between male and female aggression, but one potential contributing factor could be the general assumption that women, unlike men, do not engage in direct aggression (such as physical assault, threats of harm, etc.) (Denson et al., 2018). However, several exceptions exist to warrant a re-examination of this assumption. Aggression is a common symptom of many psychiatric diseases (Anderson, 2004; Zdanys et al., 2007; Arighi et al., 2012; Fisher et al., 2014; Gotovac et al., 2016; Lukiw and Rogaev, 2017). Diseases such as major depressive disorder (Gulland, 2016), anxiety disorders (McLean et al., 2011), postpartum psychosis (Siegel et al., 1983), post-traumatic stress disorder (PTSD) (Ditlevsen and Elklit, 2012), and dementia, affect women at significantly higher rates than men (Derreberry and Holroyd, 2019). Though PTSD is commonly associated with men, particularly those who have endured trauma because of military combat (Ditlevsen and Elklit, 2012; Crum-Cianflone and Jacobson, 2014), women have a two-fold higher risk of experiencing PTSD after a traumatic experience than their male counterparts (Ditlevsen and Elklit, 2012; Crum-Cianflone and Jacobson, 2014). In addition, instances of hyper-aggression involving direct physical attacks have also been documented in women (Lindberg et al., 2009). These observations strongly suggest that a comprehensive understanding of the neurobiology of aggressive behavior will not be possible by just focusing on male aggression.

In fruit flies, elevated female aggression has been observed under conditions of social isolation (Ueda and Kidokoro, 2002), mating (Bath et al., 2017, 2018, 2021), or nutrient scarcity (Lim et al., 2014). In addition, small populations of neurons have been identified in the fruit fly female brain, whose activation promoted very high levels of female aggression (Palavicino-Maggio et al., 2019b; Schretter et al., 2020). These results suggest that the fruit fly model of *Drosophila melanogaster* is a great model for studying female aggression. Findings from this model may provide fundamental insights about the importance of aggression in female fitness and the circuit logic by which female aggression is governed. With the availability of central brain connectomic data (Scheffer et al., 2020) and automated aggression analysis using machine vision (Schretter et al., 2020) combined with the strengths of fruit fly model, it is only a matter of time before our understanding of female aggression is significantly advanced along with male aggression.

## *Drosophila melanogaster*: A Model for Studying Aggression

**FIGURE 1:**
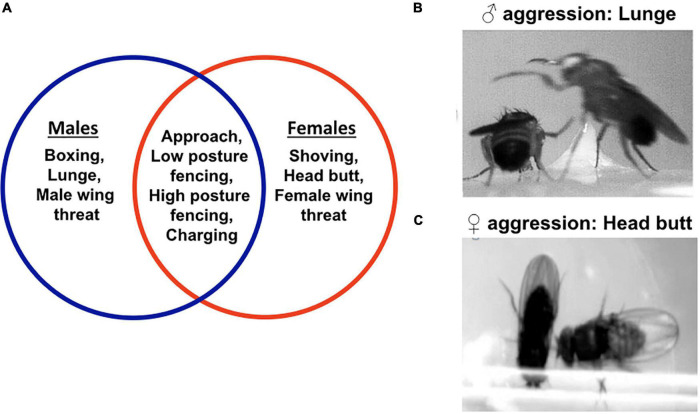
Motor programs used in fruit fly aggression. The Venn diagram shows the different motor programs used in male and female aggression. Some of the motor programs are sexually dimorphic. The motor programs encircled in blue are specific for male aggression. The motor programs encircled in red are specific to female aggression. The intersection enlists the motor programs common to both male and female aggression **(A)**. The most consistent motor program in male aggression is a lunge **(B)**. In a lunge, a male fly stands on its hind legs and snaps down on its opponent. The most consistent motor program in female aggression is a head butt. In a head butt, the female fly extends her torso and strikes the opponent with her head **(C)**.

Both male and female fruit flies exhibit aggression, and they do so by using a variety of stereotyped motor programs (Chen et al., 2002; Asahina, 2017). These motor programs are well characterized, easily recognizable and highly quantifiable, allowing researchers to perform quantitative aggression assays and study changes in aggression intensity. Male aggression in *Drosophila melanogaster* can be exhibited by different motor programs such as fencing, wing threat, lunge, boxing, tussling, holding, etc. (Chen et al., 2002). Such an extensive repertoire of motor programs probably helps the fruit fly in adapting its fighting strategy to an ever-changing set of conditions. However, not all the motor programs occur at similar frequencies in a fight suggesting that the context behind each of them is probably different (Chen et al., 2002). For example, the motor program of “boxing” which involves two male flies striking at one another with their front legs, rarely occurs in a fight (Chen et al., 2002; Sengupta et al., 2022). In contrast, the motor program of “lunge,” which involves a male fly standing on its hind legs and snapping down on its opponent, is most consistently used in intermale fights (Figure 1B; Chen et al., 2002). Similarly, female aggression uses many motor programs such as wing threat, head butt, high-posture fencing, shove, etc. (Figure 1A). The motor program of “head butt” is most consistent in female aggression (Figure 1C; Sturtevant, 1915; Nilsen et al., 2004; Palavicino-Maggio et al., 2019b), and is executed by a *Drosophila* female extending her torso and striking the conspecific with her head. To analyze changes in aggression intensity, some studies count the frequency of lunges or head butts within a given observation period, some studies count the total number of agnostic motor programs within a given observation period, while some count the percentage of animal pairs exhibiting aggression (Dierick and Greenspan, 2007; Asahina et al., 2014; Koganezawa et al., 2016; Asahina, 2017; Palavicino-Maggio et al., 2019b). In addition, the length of observation period, as well as aggression chamber setups have also varied greatly among studies (Kravitz and Fernandez, 2015). While all the aggression paradigms are correct, the differences among them are likely to influence aggression outputs and therefore, must be kept in mind before comparing results among studies. Other advantages of the fruit fly model include (a) a relatively simple brain (∼ 100,000 neurons compared to ∼ 100 billion in humans), (b) advanced genetic and molecular toolkit, (c) a genome with 60% homology to humans, and (d) availability of the hemibrain connectome (Venken et al., 2011; Scheffer et al., 2020; Raji and Potter, 2021). Despite the dissimilarities with the mammalian brain in shape and size, the fruit fly central nervous system shares many similarities with its mammalian counterpart in circuit organization, kinds of neuromodulators used, and mechanisms of neuromodulator storage, release, and recycling (Leyssen and Hassan, 2007; Yamamoto and Seto, 2014). Therefore, findings from the fruit fly model reveal at least some of the general principles of the neuromodulatory basis of aggression in mammals.

## Serotonin

The monoamine serotonin (5-HT) has been historically linked to aggression in a wide range of species including humans (Coccaro, 1989; Kravitz, 2000; Krakowski, 2003; Manuck et al., 2006). For many years, neurochemical and pharmacological studies upheld a view of a negative association between 5-HT functioning and aggression. This was based on several findings that detected low levels of 5-HT’s metabolic product 5-HIAA (5-hydroxyindoleacetic acid) in cerebrospinal fluid of military men with personality disorders, men with violent suicidal tendencies or arsonists (Brown et al., 1979; Linnoila et al., 1983; Virkkunen et al., 1987; O’Keane et al., 1992). However, research in the last decade using sophisticated genetic and pharmacological tools in the invertebrate model of *Drosophila melanogaster* presented a different view. In male fruit flies, increasing and decreasing 5-HT signaling by pharmacological interventions increased and decreased male aggression, respectively (Dierick and Greenspan, 2007). Similar trends were observed upon genetically activating and inactivating 5-HT neurons en masse in brains of male flies (Dierick and Greenspan, 2007; Alekseyenko et al., 2010). First, these observations suggest that the 5-HT system regulates male aggression in both humans and fruit flies. Second, the apparent discrepancy between the valence of the 5-HT signal and aggression outputs in humans and fruit flies suggests that 5-HT’s role in aggression regulation is complex and could be potentially influenced by multiple factors such as, magnitude of change in 5-HT signal, brain region involved, downstream 5-HT receptor cascades used, etc. Alternatively, it is also possible that 5-HT’s aggression regulatory role is different between humans and fruit flies. Additional research is required to experimentally evaluate these possibilities.

The fruit fly nervous system has ∼100 serotonergic neurons (Alekseyenko et al., 2013) and the 5-HT system has been implicated in many behaviors in addition to aggression (such as memory, circadian rhythm, courtship, etc.) (Yuan et al., 2005; Becnel et al., 2011; Sitaraman et al., 2012). To characterize a 5-HT mediated aggression-specific neural circuitry, Alekseyenko et al. (2014) used intersectional genetics (Dionne et al., 2018) and identified a pair of serotonergic neurons with aggression regulatory roles. These neurons were located in the posterior lateral protocerebrum of the central brain (5-HT PLP neurons), a region known to receive visual input from the optic lobe (Pereanu et al., 2010). Activation and inhibition of the 5-HT PLP pair of neurons increased and decreased aggression in male fights, respectively (Alekseyenko et al., 2014). Interestingly, activity manipulation of the 5-HT PLP pair also had mild effects on few other behaviors (Alekseyenko et al., 2014). For example, inactivation of the 5-HT PLP pair of neurons mildly reduced the total amount of sleep per 24 h but did not affect the social behavior of courtship. Locomotion deficits were observed upon activation as well as inactivation of the 5-HT PLP pair of neurons. Since an increased aggression phenotype as well as a decreased aggression phenotype were recorded in fruit fly males with mild locomotion deficits, it is likely that the 5-HT PLP pair’s modulatory role on aggression and locomotion are independent of each other. Since a single neuron can receive contacts from many different pre- and post-synaptic neurons (Klaassen et al., 1998), it can potentially influence multiple behaviors by activating non-overlapping circuit partners. Future experiments are required to determine whether this is the case with the 5-HT PLP pair of neurons.

A total of five 5-HT G protein-coupled receptors have been characterized in the fly model (5-HT_1A_, 5-HT_1B_, 5-HT_2A_, 5-HT_2B_, and 5-HT_7_) (Blenau et al., 2017) and a few of them have already been reported to modulate aggression. For example, the aggression-promoting 5-HT PLP pair of neurons were found to make putative synaptic contacts with 5-HT_1A_ receptor neurons, implicating the latter’s involvement in aggression (Alekseyenko et al., 2014). Another study identified two types of 5-HT_1A_ receptor neurons with opposing effects on intermale aggression (Alekseyenko et al., 2019). One of the 5-HT_1A_ receptor neurons was GABAergic, and its activation reduced aggression. The other 5-HT_1A_ receptor neuron was cholinergic, short neuropeptide F receptor^+^ (sNPFR^+^) and resistant to dieldrin GABA receptor^+^ (RDL-GABA^+^). Activation of this neuron increased aggression. Interestingly, the dendritic fields of both these neurons innervated the LC12 optic glomerulus of the ventrolateral protocerebrum, raising the possibility that visual cues input into the aggression circuit through the GABAergic and cholinergic 5-HT_1A_ receptor neurons.

While it has been shown that females with low serotonin levels exhibit higher aggression (Westergaard et al., 1999; Kästner et al., 2019), systematic studies on the role of 5-HT on aggressive behavior in all model systems have primarily focused on males. Continued examination of different components of the serotonergic system is necessary to comprehensively understand its bearing on aggression in both males and females.

## Dopamine

Like 5-HT, Dopamine (DA) has also been shown to regulate aggression in vertebrate and invertebrate models (Ryding et al., 2008; Seo et al., 2008; Alekseyenko et al., 2013). Release of DA from the nucleus accumbens has been correlated with increased aggression in rats (Van Erp and Miczek, 2000). Activation of the ventral tegmental area (VTA) dopaminergic neurons has been shown to increase isolation-induced aggression in mice (Yu et al., 2014). Another study by Mahadevia et al. (2021) identified a subgroup of the VTA dopaminergic neurons that selectively projected to the lateral septum, and whose activity was necessary for maintaining baseline aggression in mice.

The fruit fly has ∼ 125 DA neurons in each brain hemisphere (Xie et al., 2018). Blocking synaptic transmission from DA neurons en masse generated hyperactive flies that displayed increased locomotion, and rarely engaged in either courtship or aggression (Alekseyenko et al., 2010). Using intersectional genetics, Alekseyenko et al. (2013) identified two pairs of morphologically distinguishable DA neurons in the fly brain with aggression regulatory roles: the tritocerebral neurons (T1) and the protocerebral posterior medial 3 (PPM3) neurons. Activating and inactivating the T1 and PPM3 pairs of neurons enhanced male aggression without any major effect on the behavior of locomotion (Alekseyenko et al., 2013). These observations suggest (i) activity manipulation of the T1 and PPM3 pairs of neurons has selective effects on aggression, (ii) relationship between DA signaling and aggression is not linear, with higher or lower amounts of DA-signaling resulting in an increase in aggression intensity. This kind of relationship is also known as the “U-shaped relationship.” A similar U-shaped relationship has been reported for DA and spatial working memory in the rodent model, with increased and decreased DA signaling in the prefrontal cortex inducing working memory deficits (Zahrt et al., 1997; Cools and D’Esposito, 2011). While these findings indicate a need for maintaining an optimal concentration of basal DA for cognitive functions such as aggression or working memory, the mechanistic basis of U-shaped effect is largely unknown.

Pharmacological studies in the murine models have highlighted the involvement of DA receptors in aggression regulation. Indeed, the current therapeutic interventions for treatment of aberrant aggression include antagonists of different DA receptors (Kudryavtseva, 2005; Khushu and Powney, 2016). For example, Haloperidol, which is primarily a D2-receptor antagonist, has been routinely used to treat violent behavior in aggressive patients, especially those suffering from psychosis (Nelson and Trainor, 2007; Khushu and Powney, 2016). However, the administration of these drugs is often complicated by negative side effects such as sedation, metabolic disorders, and tardive dyskinesia (de Almeida et al., 2005; Nelson and Trainor, 2007; Palavicino-Maggio and Kuzhikandathil, 2016), suggesting modulation of different biological processes through multiple site receptor-action. Therefore, though DA system has been shown to be necessary for aggression, details of the circuit mechanisms through which DA and its receptors specifically modulate aggression, remain largely unknown. In *Drosophila*, four G-protein coupled receptors (Dop1R1, Dop1R2, DD2R, and DopEcR) have been identified (Yamamoto and Seto, 2014). Of them, Dop1R1 has been found to regulate different types of arousal states: it positively regulates sleep-wake transitions (a form of endogenous arousal) and negatively regulates startle-induced arousal (a form of exogenous arousal) (Lebestky et al., 2009). In Alekseyenko et al. (2013), the presynaptic endings of dopaminergic T1 intermingled with the DD2R neurons in the protocerebral bridge and that for PPM3 neurons intermingled with the Dop1R1 neurons in the fan-shaped body and noduli of the central complex. While this raised the possibility that aggression regulatory T1 and PPM3 neurons interacted with DD2R and Dop1R1 receptors as downstream targets, direct experimental evidence demonstrating DD2R’s and/or Dop1R1’s involvement in aggression is still lacking.

Studies researching the function of DA in female aggression have been predominantly described in the context of courtship. Often when mated, immature, or older females come into encounter with a courting male, they may engage in pre-or post-mating female aggression that contains defensive aggressive behavior such as fleeing, kicking, and shoving, indicating rejection (Speith, 1952; Manning, 1966; Connolly and Cook, 1973; Ueda and Kidokoro, 2002; Sakurai et al., 2013; Bontonou and Wicker-Thomas, 2014; Bussell et al., 2014; Zhou et al., 2014; Li et al., 2016; Bath et al., 2017, 2018). Dopaminergic inputs have been noted to drive this circuit and govern female receptivity behavior (Zhou et al., 2014; Rezával et al., 2016; Ishimoto and Kamikouchi, 2020). This circuit is comprised of (R) neurons found in the ellipsoid body of the central brain (Martín-Peña et al., 2014; Omoto et al., 2018); PPM3 transmits DA specifically to R4d neurons, and activation of these neurons has been demonstrated to prolong the duration of this type of defensive behavior (Ishimoto and Kamikouchi, 2020).

The DA system, just like any other neuromodulator, is complex with regulatory roles in many behaviors. With more than 100 DA neurons sending arbors to different parts of the brain, the same brain region potentially generating paradoxical effects of activation or inactivation of downstream circuit depending on the DA receptors used, and a non-linear relationship between DA and male aggression at least in the fruit fly model, understanding the specifics of DA’s aggression regulatory role is not straightforward. Nevertheless, findings from the fruit fly model provide relevant entry points into unraveling DA’s regulatory roles in both male and female aggression.

## Octopamine

Noradrenaline (NA) has been implicated in mammalian aggression (Yanowitch and Coccaro, 2011). Research in male mice indicated that perturbation of the NA signaling reduced aggression (Marino et al., 2005). The invertebrate ortholog of noradrenaline is Octopamine (OA). There are about ∼ 100 OA neurons in fruit fly brain (Busch et al., 2009; Farooqui, 2012). In the fruit fly model, OA is necessary for maintaining baseline aggression in both males and females (Zhou et al., 2008). Almost all the studies investigating how OA deficiency affects fly aggression used a deletion-mutant of *Tyramine ß-hydroxylase* gene (*Tßh^nM18^*) (Hoyer et al., 2008), that encodes a key biosynthetic enzyme in OA synthesis. *Tßh^nM18^* males performed reduced lunges and increased male-male courtship toward conspecific males (Certel et al., 2007). *Tßh^nM18^* females performed reduced number of head butts in female-female pairings (Zhou et al., 2008). These observations suggest OA signaling regulates aggression in both male and female fruit flies. Subsequent reports investigating the role of OA signaling in aggression focused on male aggression. Four kinds of OA-receptors (OAMB, Octβ1R, Octβ2R, and Octβ3R) have been characterized in *Drosophila* (El-Kholy et al., 2015). Watanabe et al. (2017) found that, OAMB receptor neurons labelled by a *GAL4* driver made from the cis-regulatory element of the *OAMB* gene (*R47A04-GAL4*), resulted in decreased aggression and increased courtship in male-male encounters. Altogether, these reports suggest that OA-signaling regulates appropriate behavioral choices in males.

In *Drosophila*, as in most species, males court females as potential mates and never attack them. Male-male pairings, on the other hand, are characterized predominantly by aggression with little or no courtship (Fernández et al., 2010; Wang et al., 2011; Monyak et al., 2021; Sengupta et al., 2022). In that regard, the increased courtship and decreased aggression phenotypes of the *Tßh^nM18^* intermale fights could likely result from aberrant sex recognition. One of principal sensory modalities guiding sex-recognition and behavioral decisions in fruit flies is its pheromone system (Fernández and Kravitz, 2013). Indeed, elimination of some of the male-enriched pheromones such as (*z*)-7-tricosene (7-T), results in reduced aggression and increased courtship in male-male encounters (Wang et al., 2011). Sensory neurons expressing the chemoreceptor gene *Gr32a* (Gr32a neurons) have been identified to mediate the behavioral effects of 7-T. Mutant males lacking *Gr32a* (*Gr32a* −/−) or males with ablated Gr32a neurons have been shown to phenocopy the decreased aggression and increased intermale courtship behaviors of the *Tßh^nM18^* males (Wang et al., 2011; Andrews et al., 2014). These results suggest that sex-dependent pheromonal inputs processed by sensory Gr32a neurons are transduced upstream at least by the OA system for maintaining the appropriate balance of aggression and courtship in male-male pairings. Consistent with these findings, axons of Gr32a neurons have been found to make putative synaptic contacts with OA neurons in the suboesophageal ganglion (Andrews et al., 2014).

The above section suggests that OA signaling regulates aggression by modulating the pheromone-brain axis in fruit flies. A recent study (Jia et al., 2021) showed that OA signaling could also regulate aggression by modulating the gut-brain axis. The microbiome, a collection of microbes such as bacteria, archaea, fungi, and viruses, inhabit almost all the exposed surfaces of the body, with humans having the greatest density in their gastrointestinal tract or gut (Hsu et al., 2019). Using the fruit fly model, Jia et al. (2021) showed that gut microbiome selectively promoted both male and female aggression using OA neuromodulation. Germ-free males exhibited reduced aggression and a concomitant downregulation in OA signaling. Apart from a reduced expression in two major genes of the OA biosynthesis pathway, *Tyrosine Decarboxylase 2* (*Tdc2*) and *Tyramine ß-Hydroxylase* (*Tßh*), the germ-free males also displayed reduced Tdc2 immunoreactivity in subsets of OA neurons in the central brain. An interesting question is, how are signals from the gut transmitted to OA neurons in the central brain?

Does an enhancement of OA signal increase aggression in *Drosophila* males? Enhancing OA signaling in the less aggressive group-housed flies, by either feeding them the OA agonist Chlordimeform (CDM) or genetically overexpressing the *Tßh* gene, increased aggression (Zhou et al., 2008). But the same treatment was unable to raise the intensity of aggression among more aggressive socially naive males (Zhou et al., 2008). One hypothesis is, under normal conditions neural circuits are already saturated with OA signaling in socially naïve males and thus, any further enhancement of OA signaling does not result in an increase in aggression intensity. It would be interesting to overexpress the OA receptors in socially naïve flies and subsequently test the effect of enhancing OA signaling on aggression. It is worth mentioning that OA feeding alone did not increase aggression in group-housed flies in another study but did increase aggression upon *OAMB* overexpression in *R47A04-GAL4* neurons (Watanabe et al., 2017). The discrepancy in the observed outputs of aggression intensity could potentially result from different feeding strategies, chamber set ups and/or aggression scoring protocols.

## Acetylcholine

The neuromodulatory role of Acetylcholine (ACTH) in aggression was initially suggested in the 1970s when ACTH-treated animal models revealed variation in aggression levels (Bandler, 1969; Silverman, 1969, 1971; Igić et al., 1970; Allikmets, 1974). Furthermore, known acetylcholine receptors (AchRs), nicotinic (nAchRs) and muscarinic (mAchRs) receptors also have been implicated in aggression regulation (Bandler, 1969, 1970; Berntson et al., 1976; Picciotto et al., 2015). Male and female cats, for example, exhibited aggressive behaviors in response to cholinergic agonists; however, muscarinic antagonists inhibited aggression. Nicotine and other nAChR-targeting drugs have been shown to reduce aggression in animal models (Bandler, 1969, 1970; Igić et al., 1970; Driscoll and Baettig, 1981; Yoburn and Glusman, 1984).

ACTH is found in many excitatory synapses in the *Drosophila* central nervous system (Buchner, 1991; Shih et al., 2019). Studies suggest that fruit fly has ten nAChRs and three mAChRs (Su and O’Dowd, 2003; Collin et al., 2013; Ren et al., 2015; Silva et al., 2015). While it is believed that nAChRs mediate fast excitatory synapses currents, mAChRs have been discovered to function as both excitatory and inhibitory modulators (Collin et al., 2013; Ren et al., 2015; Bielopolski et al., 2019).

It is possible that cholinergic signaling has opposing behavioral effects in both males and females. Enhanced female chasing, aggression, and territorial behavior, for example, were discovered upon activating *R26E01-GAL4* labeled neurons (McKellar and Wyttenbach, 2017; Palavicino-Maggio et al., 2019b). An intersectional study further revealed that neurons in the female fly brain’s pC1 region (pC1α neurons) were cholinergic, expressed female isoform of the sex determination gene *doublesex* (*dsx*), and were responsible for this behavior (Palavicino-Maggio et al., 2019b). Other studies found another subset of neurons, known as the aIPg neurons (Cachero et al., 2010) that were also cholinergic and expressed sNPF, implying that an excitatory neural network regulated female aggression as well (Schretter et al., 2020). Both aIPg and pC1 cholinergic clusters have been found to mediate female aggression, with activation promoting persistent aggressive behavior and inhibition reducing aggression (Palavicino-Maggio et al., 2019b; Deutsch et al., 2020; Schretter et al., 2020; Chiu et al., 2021). Furthermore, additional research discovered that cholinergic neurons in the pC1 circuit also facilitate female receptivity during courtship behavior (Zhou et al., 2014; Rezával et al., 2016). The extent to which acetylcholine in neurons regulates female aggression and how this regulation coincides with that of mating behavior remains unknown.

In contrast, it has been found that blocking a single cholinergic neuron increases aggression in males (Alekseyenko et al., 2019), and feminizing cholinergic neurons in male brains similarly alters aggression (Mundiyanapurath et al., 2009). The brains of fruit flies include many cholinergic neurons, many of which are in areas that provide sensory information to the central brain (Kitamoto et al., 1995; Yasuyama and Salvaterra, 1999; Salvaterra and Kitamoto, 2001; Olsen et al., 2007). The detailed mechanism by which cholinergic neurons regulate aggression in males and females is unknown, and this has raised several questions, such as (i) are female cholinergic neurons distinct from the male cholinergic neurons? (ii) are there morphological distinctions amongst neuronal arbors? (iii) is there any variation in the quantities of acetylcholine or the transmitter release machinery?

## Sex Peptide

Seminal proteins have been found to have a sexually dimorphic effect on female and male behavior in both vertebrates and invertebrates (Cooke et al., 1998; Heifetz and Wolfner, 2004; Wigby and Chapman, 2005; Yapici et al., 2008; Yang et al., 2013; Lee et al., 2015; Garbe et al., 2016; Asahina, 2018; Isaac, 2019).

In fruit flies, mating has been shown to modulate female aggression, suggesting a link between neural circuits of mating and aggression (Nilsen et al., 2004; Bath et al., 2017). During copulation, the male’s seminal fluid delivers a sex peptide (SP) (Chen et al., 1988; Aigaki et al., 1991; Chapman et al., 2003; Liu and Kubli, 2003; Heifetz and Wolfner, 2004; Feng et al., 2014), which activates SP receptors expressed in sex peptide sensory neurons that connect post-synaptically to the sex peptide abdominal ganglion (SAG) neurons (Yapici et al., 2008; Häsemeyer et al., 2009; Yang et al., 2009; Rezával et al., 2012; Bath et al., 2020). According to one study, female *Drosophila* mated with older males exhibit lower aggression reflecting changes in sex peptide activation (Bath et al., 2020).

SAG neurons have also been shown to be female-specific and implicated in post-mating behavior (Feng et al., 2014; Wang et al., 2020). Anatomical studies have shown that axons of SAG neurons project directly to the central complex of the brain (Feng et al., 2014; Wolff et al., 2015; Wang et al., 2020), ipsilaterally into the flange (periesophageal) area, and bilaterally into the superior medial protocerebrum, which includes the pars intercerebralis (PI) (Wang et al., 2020). Intrinsically, PI is a mammalian hypothalamus homolog that governs many processes, including sleep, alertness, locomotor cycles, aggression, and eating (De Velasco et al., 2007; Erion et al., 2012; Cavanaugh et al., 2014; Davis et al., 2014; Barber et al., 2016). However, the significance of SAG neurons in female aggression, is unknown.

**FIGURE 2:**
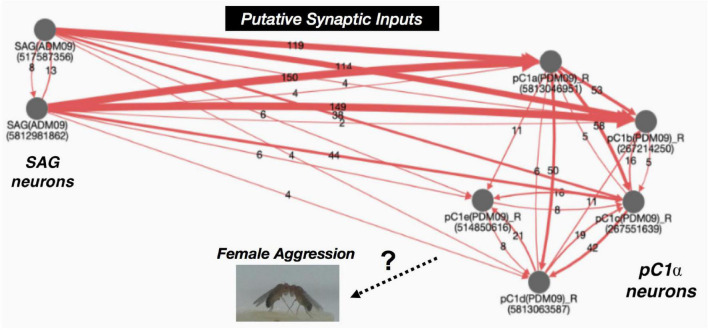
Connectivity graph of SAG neurons and pC1α neurons. SAG neurons project major inputs into the pC1α neurons. pC1α neurons make reciprocal connections within the pC1 neuronal cluster. Red lines indicate synaptic connections and the numbers within the arrows represent number of shared synapses. Arrows indicate putative target. Numbers underneath traced neurons indicate the ID number from the neuPrint server (https://neuprint.janelia.org/) (Zheng et al., 2018; Scheffer et al., 2020; Wang et al., 2020).

Interestingly, in the pC1α activated female aggression study (Palavicino-Maggio et al., 2019b), *dsx* labeling was identified in the abdominal ganglion area, which also contains SAG neurons. According to electron microscopy (EM) data analysis (Figure 2) and other studies, SAG neurons project a vast number of putative synaptic input connections to pC1α neurons (pC1a-pC1e) (Zheng et al., 2018; Schretter et al., 2020; Wang et al., 2020). Neuronal tracings also revealed pC1α neurons have reciprocal connections within the pC1α neuronal cluster (Figure 2). Given that SAG neurons provide a significant number of synaptic inputs to pC1α neurons, is it possible that SAG neurons also regulate female aggression? This still remains an open question.

## Tachykinin

Tachykinins (Tk) constitute a group of evolutionary conserved neuropeptides present in both vertebrates and invertebrates wherein they perform a multitude of functions in controlling behavior, physiology and development (Jiang et al., 2013; Nässel et al., 2019). Substance P, a member of the Tk family has been linked to aggression-induction and regulation in many studies (Bhatt et al., 2003; Katsouni et al., 2009). Genetically knocking-out its potent Tk receptor, *Neurokinin-1 Receptor* (*NK1*) in the mice model has been reported to reduce aggression in resident-intruder experiments and alter nociceptive reflexes and analgesia (De Felipe et al., 1998). How tachykinins regulate aggression levels has been systematically studied in the fruit fly model (Asahina et al., 2014). Activation of a subset of male-specific Tk neurons (*Tk-GAL4^FruM^*) robustly increased male aggression, while their silencing reduced aggression. Immunostaining experiments revealed that *Tk-GAL4^FruM^* neurons expressed acetylcholine in addition to the neuropeptide Tk, thereby suggesting that acetylcholine may play an additional role in this circuit. A deletion mutation in the *Tk* gene in the homozygous form significantly reduced aggression, a phenotype that was rescued by expressing Tk neuropeptide in the Tk neurons. This suggests that at least part of the aggression modulatory function of the Tk neurons is mediated by the Tk peptides. *Drosophila* tachykinin has two known receptors: Tachykinin-like receptor 86C (TakR86C) and Tachykinin-like receptor 99D (TakR99D) (Birse et al., 2006; Poels et al., 2009; Pavlou et al., 2014). Owing to differential sensitivity to ligand Tk (Asahina et al., 2014, Asahina, 2017), both these receptors have been postulated to have non-overlapping roles in aggression regulation. The TakR99D receptor has a higher sensitivity to Tk and is postulated to mediate baseline aggression. TakR86C is postulated to regulate transient, intense bursts of aggression as seen during thermogenetic activation of *Tk-GAL4^FruM^* neurons (Asahina et al., 2014; Asahina, 2017). Future investigations delineating how and which TakR99D and TakR86C receptor neurons interact with the Tk-GAL4*^FruM^* neurons are necessary to characterize the circuit-mechanisms involved therein.

*Tk-GAL4^FruM^* neurons are specified in males by the *fruitless* (*fru*) gene, a central component of the sex determination pathway (Wohl et al., 2020). Transcripts from the P1 promoter of the *fru* locus are spliced differently in males and females (Demir and Dickson, 2005). By gene-targeting, *fru* alleles *fru^M^* and *fru^F^* have been generated, which force male-specific and female-specific P1 *fru* splicing in females and males, respectively. The resulting *fruM* females are said to be masculinized, and the resulting *fruF* males feminized (Demir and Dickson, 2005). *Tk-GAL4^FruM^* neurons are absent in wild type females but are present in the *fruM* females, where they are comparable to their male counterpart in number and morphology (Asahina et al., 2014; Wohl et al., 2020). Strikingly, optogenetic activation of the *Tk-GAL4^FruM^* neurons in *fruM* females induced the male-specific motor program of “lunge,” albeit at a low frequency, against wild type females or feminized males. *Tk-GAL4^FruM^* activation did not induce female-specific “head butts” in these fights. Since lunge is a male-specific fighting pattern, these observations argue that *Tk-GAL4^FruM^* neurons are a part of a neural circuit whose activation is sufficient for releasing significant amounts of male-patterns of aggression in females.

An intriguing question in the field of behavioral neuroscience is, how are male and female patterns of aggression encoded in the brain? In the fruit fly model, several features of male and female aggression are sexually dimorphic. Some of the motor programs used in male aggression, such as lunge and boxing, are male-specific (Figure 1B; Sturtevant, 1915; Hoffmann, 1987; Chen et al., 2002; Nilsen et al., 2004; Palavicino-Maggio et al., 2019a) and some in female aggression such as head butt, shove, are female-specific (Figure 1C; Manning, 1960; Ueda and Kidokoro, 2002; Nilsen et al., 2004; Palavicino-Maggio et al., 2019b). Social hierarchy or “dominance,” a condition in which the dominant fly retains possession of the resources (such as food and territory) to the exclusion of the subordinate conspecific, is frequently established in most male fights but not in female fights (Chen et al., 2002; Nilsen et al., 2004; Palavicino-Maggio et al., 2019b). In addition, females often share resources during fights, unlike their male counterparts (Chen et al., 2002; Nilsen et al., 2004). What genes and neurons encode the sex-specific differences in aggression? A previous study found that *fruM* females and *fruF* males fought using significant amounts of male and female patterns of aggression, respectively (Vrontou et al., 2006). These results suggest that the sex-specific differences in fruit fly aggression are genetically encoded by at least the *fru* gene. Findings from Wohl et al. (2020) further refine our understanding by identifying a small group of male-specific neurons *Tk-GAL4^FruM^* whose activation induced male patterns of aggression in masculinized females. Overall, these results provide an important framework on which to further research the neurobiological determinants of sexual dimorphism of aggression.

## Neuropeptide F

Neuropeptide Y (NPY) is a 36-amino acid peptide that belongs to the NPY family of peptides along with peptide YY (PYY) and pancreatic polypeptide (PP). NPY is expressed widely in the mammalian brain and functions through five known G-protein coupled receptors Y1, Y2, Y4, Y5, and Y6. The NPY system regulates feeding, energy, homeostasis, stress, etc. (Reichmann and Holzer, 2016; Huang et al., 2021). NPY has also received a lot of attention because of its anxiolytic properties that are primarily mediated by Y1 receptor activation (Karlsson et al., 2008; Reichmann and Holzer, 2016). Perhaps not surprisingly, increased territorial aggressive behavior was reported in *Y1* knockout mice (Karl et al., 2004). NPY has been identified in invertebrates including fruit flies where it is called NPF to reflect the change from tyrosine (Y) to phenylalanine (F) in the C-terminal end (Fadda et al., 2019). To probe the role of NPF in aggression using the fruit fly model, Dierick and Greenspan (2007) genetically perturbed NPF signaling by blocking synaptic transmission from NPF neurons labeled by an *NPF-GAL4* driver. Compared to parental controls, a higher percentage of males engaged in aggressive interactions when synaptic transmission from *NPF-GAL4* neurons was blocked (Dierick and Greenspan, 2007). In contrast, another study detected a slight increase in aggression upon thermogenetic activation of the *NPF-GAL4* neurons (Asahina et al., 2014). These results possibly point toward the necessity of an optimal level of NPF signaling for maintaining baseline aggression, an effect also seen with DA (Alekseyenko et al., 2013). In other words, a U-shaped relationship potentially exists between the NPF signal and aggression. However, it is worth mentioning that the chamber set-ups, as well as aggression scoring parameters, were vastly different between Dierick and Greenspan (2007) and Asahina et al. (2014). Additional experiments may have to be performed before directly comparing results from the two studies.

The *NPF-GAL4* labels ∼ 30 neurons that extend their neuronal processes throughout the central brain and VNC, and at least some of these cells are male-specific (Shao et al., 2017). The next question is, are all or a specific subpopulation of the NPF neurons required for increasing aggression? Dierick and Greenspan (2007) expressed the female-specific *Transformer* gene in the *NPF-GAL4* labeled cells (*NPF-GAL4/UAS-TRA*) to eliminate *NPF* expression in the male-specific NPF cells. Interestingly, *NPF-GAL4/UAS-TRA* males recapitulated the aggression-inducing phenotype of the synaptically blocked *NPF-GAL4* neurons (Dierick and Greenspan, 2007). This result raises the possibility that male specific NPF neurons were regulators of baseline aggression. NPF is known to bind to a single receptor NPF receptor (NPFR) (Chung et al., 2017). Presently, we do not know which NPFR neurons synaptically connect to NPF cells to regulate aggression. Next, we also do not know how NPF system regulates female aggression. Since *NPY* deletion has been found to increase depressive behaviors in female mice (Nahvi and Sabban, 2020; Nahvi et al., 2021), similar to its male counterparts, it would be interesting to investigate how genetically and/or pharmacologically manipulating the NPY/NPF system influence female aggression.

## Drosulfakinin

Neuropeptide Cholecystokinin (CCK) has been studied extensively for its anxiogenic effects. It is synthesized as a 115 amino acid preprohormone which is proteolytically cleaved to generate many biologically active peptides (Netto and Guimarães, 2004; Bowers et al., 2012). Administration of CCK tetrapeptide (CCK-4) induced panic attacks in humans (Bradwejn, 1993). RNA interference (RNAi) mediated knockdown of *CCK* in the VTA of mice resulted in manic-like phenotypes (Arey et al., 2014). CCK is also present in the gastrointestinal tract (GI) where it regulates many important GI functions such as satiety and food ingestion (Moran, 2000; Moran and Kinzig, 2004). The *Drosophila* ortholog of *CCK* is called *Drosulfakinin* (*Dsk*), and its modulatory role has been studied in male sexual arousal (Wu et al., 2019) and satiety (Nässel and Williams, 2014). In *Drosophila*, the *Dsk* gene encodes three mature peptides: Dsk 0, Dsk1, and Dsk2. Of these, Dsk1 and Dsk2 are known to be CCK-like peptides. Two G-protein coupled receptors have been identified for the Dsk peptides: CCKLR17D1 and CCKLR17D3 (Nässel and Williams, 2014; Wu et al., 2019).

One of the first glimpses of Dsk’s connection in fruit fly aggression came in 2014 when Williams et al. (2014), reported octopaminergic signaling regulated male aggression by controlling Dsk expression in insulin producing cells. A more detailed analysis of Dsk’s role in aggression has come from a study by Wu et al. (2020). Genetically knocking out *Dsk* (*Dsk −/−*) reduced male aggression without interfering with locomotion or courtship (Wu et al., 2020). However, *Dsk −/−* males also exhibited increased feeding behavior (Wu et al., 2020). Genetically silencing and activating a subpopulation of ∼ 8 Dsk neurons in the fruit fly brain labeled by a *Dsk-GAL4* driver reduced and increased male aggression, respectively. These results suggest that both the Dsk molecule as well as the *Dsk-GAL4* labeled neurons are necessary for male aggression. Of the two Dsk receptors, loss-of-function mutants of *CCKLR17D1* (*CCKLR17D1-/y*) and not *CCKLR17D3* (*CCKLR17D3-/y*) recapitulated the reduced aggression phenotype of the *Dsk −/−* males (Wu et al., 2020). Moreover, the aggression-promoting effect of activated *Dsk-GAL4* neurons was lost in the *CCKLR17D1* mutant background suggesting CCKLR17D1 receptor system acts downstream to mediate the aggression promoting role of activated *Dsk-GAL4* neurons (Wu et al., 2020).

*Dsk-GAL4* neurons were reported to be synaptically connected to a subset of male-specific P1 neurons, popularly known as P1^a^, whose activation has been shown to simultaneously increase aggression and courtship in male-male pairings (Hoopfer et al., 2015). When P1^a^ neurons were activated in *Dsk −/−* males, its aggression-promoting effect was severely suppressed while its courtship-promoting effect was preserved, suggesting that the Dsk system acts downstream of activated P1^a^ neurons to promote male aggression. Overall, these research findings provide important insights into Dsk’s aggression modulatory role. However, many outstanding questions remain. The Dsk system in fruit flies, like that of mammals, is implicated in both aggression and feeding behavior and right now, we do not know whether the same or different subsets of Dsk neurons regulate feeding and aggression (Moran, 2000; Moran and Kinzig, 2004; Nässel and Williams, 2014; Wu et al., 2020). Furthermore, we also do not know whether one or both CCK-like Dsk peptides are necessary for aggression regulation.

In contrast to Wu et al. (2020), another contemporary study by Agrawal et al. (2020) reported that *Dsk* knockdown using RNAi increased social-isolation mediated aggression (Agrawal et al., 2020). Since the two investigations (Agrawal et al., 2020; Wu et al., 2020) differed in multiple aspects, it is hard to speculate on the possible reasons behind the seemingly contradictory findings. Some of these differences are as follows: (i) chamber set-ups and scoring paradigms were different making direct comparison of results difficult (ii) techniques used for reducing *Dsk* expression were different. It is possible that Dsk signaling was perturbed to different degrees in the two studies, thereby resulting in different aggression outputs (iii) use of different *Dsk-GAL4* drivers. Unlike the *Dsk-GAL4* used in Wu et al. (2020), the *Dsk-GAL4* used by Agrawal et al. (2020) targeted a group of Dsk neurons that included the Dsk^+^ insulin-like peptide Dilp2-producing neurons in the PI of the fly brain (Nichols, 1992; Nichols and Lim, 1995; Söderberg et al., 2012; Asahina et al., 2014; Agrawal et al., 2020). Interestingly, *Dsk* RNAi in the Dilp2-producing neurons using a *Dilp2-GAL4* driver also increased social-isolation mediated intermale aggression. These results suggest that reduced Dsk signaling in the PI increased intermale aggression upon social isolation. In view of the results from Agrawal et al. (2020) and Wu et al. (2020), one hypothesis is, neurons within the Dsk population exert heterogenous effects on aggression. Future investigations employing genetic mosaic techniques, such as mosaic analysis with repressible cell marker (Wu and Luo, 2006), may be used to stochastically label individual or reduced subsets of Dsk neurons and analyze their roles in aggression.

Finally, in common with most neuromodulators, Dsk’s role in female aggression is poorly understood. Wu et al. (2020) reported that *Dsk* knockout (*Dsk* −/−) suppressed female aggression. But a detailed picture of Dsk-mediated female-specific aggression circuitry is lacking. An interesting question is whether the Dsk-mediated aggression circuit in males and females involves common or sexually dimorphic set of neurons. Just like any neuromodulator, the Dsk system is complex, with links in multiple behaviors, likely by the recruitment of different peptides, receptors, and neurons. Future experiments addressing some of the questions addressed here may help understand important aspects of Dsk’s role in aggression.

## Conclusion and Future Directions

Neuromodulation constitutes a principal mechanism for generating flexible outputs of a stereotypical behavior such as aggression in both vertebrates and invertebrates. Aggression released at different intensities may be considered as flexible outputs of the behavior. Research in the fruit fly model of *Drosophila melanogaster* have made important strides in identifying specific groups of cells in the central brain system as parts of circuits whose activity manipulation changes the intensity of aggression. However, a comprehensive understanding of the circuit dynamics is lacking. In other words, finer details of how neuromodulation is achieved mechanistically are limiting. There are several ways through which neuromodulators may encode different intensities of aggression. For example, neuromodulators can coordinate multiple neuronal circuits encoding the fly’s internal and external states to compute and release aggression at a certain intensity (Bargmann, 2012; Marder, 2012). Neuromodulators can also effectively reconfigure new circuits from existing ones by recruiting new neurons or excluding current members and in doing so, alter the output intensity of aggression (Bargmann, 2012; Marder, 2012). In addition, neuromodulators can modify the excitability of an existing circuit to release aggression at an intensity appropriately matched with internal and external environments (Bargmann, 2012; Marder, 2012). At the present moment, we do not know which of these mechanisms are at work during aggression in male-male or female-female encounters. However, with the recent advances in the fly toolkit such as (i) connectomic data showing anatomical connections between different brain regions, (ii) an ever expanding genetic and molecular toolkit making precise manipulation of neuronal activity possible, and (iii) a rapidly growing set of imaging tools allowing researchers to investigate neuronal structure and function across several spatial and temporal scales, it will not be long before a comprehensive picture of the neuromodulatory basis of aggression regulation, from sensory processing to behavior computation, starts emerging.

## Author Contributions

CP-M and SS conceived and designed the review. Both authors contributed equally to the article and approved the submitted version.

## Conflict of Interest

The authors declare that the research was conducted in the absence of any commercial or financial relationships that could be construed as a potential conflict of interest.

## Publisher’s Note

All claims expressed in this article are solely those of the authors and do not necessarily represent those of their affiliated organizations, or those of the publisher, the editors and the reviewers. Any product that may be evaluated in this article, or claim that may be made by its manufacturer, is not guaranteed or endorsed by the publisher.
